# Symptomatic Unilateral Spondylolysis Associated With Nonspondylolytic Lateral Clefts in Adults: Review of an Infrequently Reported Pathology

**DOI:** 10.7759/cureus.928

**Published:** 2016-12-12

**Authors:** Vibhu Krishnan Viswanathan, Sakthivel Rajan Rajaram Manoharan, Surabhi Subramanian

**Affiliations:** 1 Neurosurgery, Ohio State University Medical Center; 2 Orthopaedic Surgery, UAB School of Medicine; 3 Radiology, Government Medical College, Nagpur

**Keywords:** laminolysis, pediculolysis, spondylolysis

## Abstract

Nonspondylolytic lateral clefts of the lumbar neural arch (laminolysis and pediculolysis) are rare pathologies that usually occur consequent to repetitive stress injuries in patients with unilateral spondylolysis. These lesions are different from the usual bilateral spondylolytic defects, and their management depends upon the chronicity and the type of bony defect. We hereby discuss the verdict of current literature on underlying pathomechanics and ideal management guidelines of these rare lesions.

## Introduction and background

Lytic spondylolisthesis typically results from a bilateral defect in the pars interarticularis, which occurs following acute fractures or stress/fatigue failures [1]. Such a defect usually exists bilaterally. However, in situations of chronic, unilateral pars lysis, the neural arch tends to fail elsewhere on the contralateral side, secondary to recurrent strains imparted across the posterior elements of the vertebra [2]. These unusual situations result in the development of defects in the nonisthmic portions of the neural arch, involving either the contralateral pedicle or lamina [3]. We hereby discuss comprehensively the pathomechanism behind these uncommon lesions and recommend the appropriate management protocols in such situations.

## Review

The earlier concept of benign nature of unilateral lytic lesions of the pars interarticularis has been controverted by recent literature reports confirming the existence of regional instabilities generated across the vertebral segments with asymmetric pars defects [4-6]. In chronic unilateral spondylolysis, the posterior vertebral elements gradually tend to yield elsewhere on the contralateral side [4]. The most common ensuing defect in such scenarios is a defect at the contralateral pars itself [4]. However, on rare occasions, the neural arch may give way at the contralateral pedicular or laminar levels under the influences of persistent mechanical stresses. These lesions have been classified as nonspondylolytic lateral clefts of lumbar vertebral arch [3].

### Classifications

Classification of nonspondylolytic clefts of the lumbar vertebral arch can be based on the anatomic location, as described in 1983 by Johansen et al. [3], and include retrosomatic (pedicle) or retroisthmic (lamina) defects. The pedicle defects were described in detail by Gunzburg et al. [2], who coined the term “pediculolysis” for chronic pedicular stress fractures. Miyagi et al. classified laminolytic defects into hemilaminar (sagittal fracture-line orientation) and interlaminar subtypes (coronal orientation) based on their morphological appearance [7]. Both these defects (pediculolysis and laminolysis) occur following a similar mode of failure as in spondylolysis, i.e., recurrent extension loading of the posterior vertebral elements with superadded rotational stresses.

### Pathomechanics

The initial description of unilateral pars lysis was made in 1953 by Stewart [4], and the clinical significance of such asymmetric lesions have remained controversial. Porter et al. [4] described the biomechanical implications of unilateral pars defects and demonstrated their association with wedging of a vertebral body, hypoplasia of the ipsilateral neural arch, rotational malalignment of spinous processes, and vertebral hemilisthesis. Under such an altered biomechanical environment, the existence of significantly increased mechanical stress elements on the contralateral neural arch (12.6-fold greater stresses) was established by the in vitro experimental model created by Sairyo et al. [6]. The vertebra tends to fail more commonly at the contralateral pars interarticularis; although rare reports of nonspondylolytic lateral vertebral arch clefts occurring in patients with contralateral pars lysis have been described.

Although acute injuries can cause such disruptions of the posterior spinal elements, the more common underlying mechanism has been reported to be chronic stress fractures. Recurrent microtrauma following repetitive hyperextension and rotational stresses delivered across a relatively hyperlordotic, hypoplastic, or dysplastic lumbar spine lead to gradual failure of the neural arch [8]. More commonly, these lesions have been described in active laborers [9-10] or adolescent sportspersons who are prone to repeated, chronic injuries [6]. Nevertheless, certain reports have also described [10] single level or multilevel insufficiency pedicle fractures in elderly patients in association with severe osteoporosis or renal osteodystrophy.

### Evaluation

**Figure 1:**
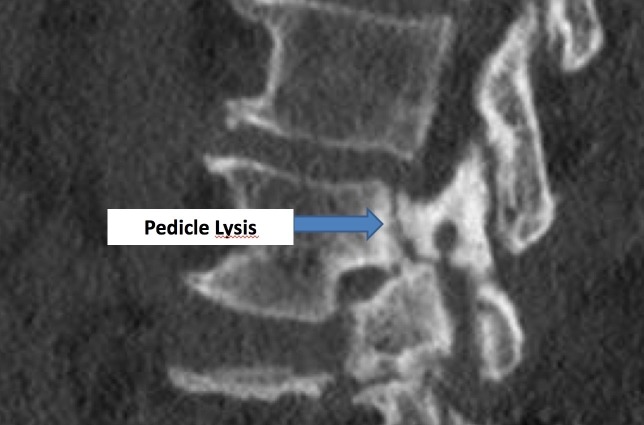
Sagittal CT image

**Figure 2:**
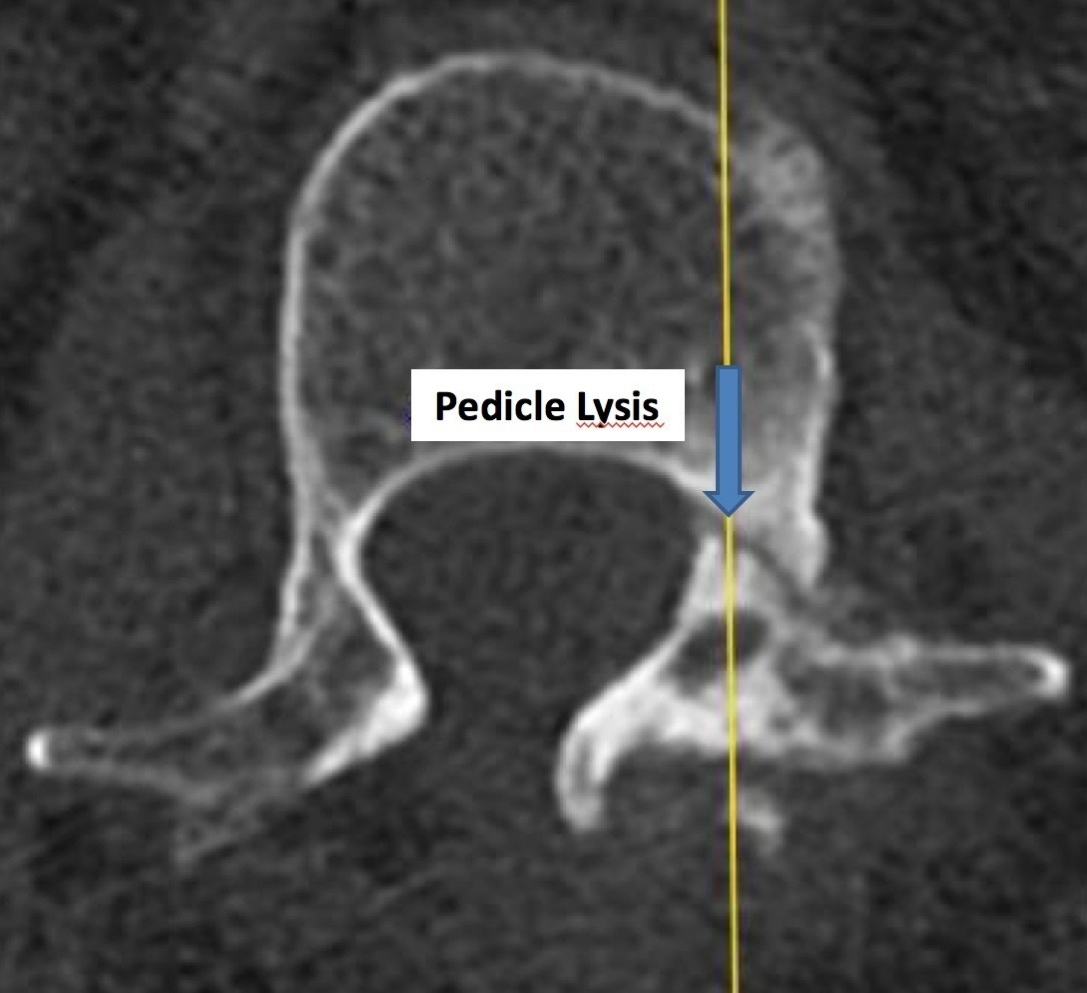
Axial CT image

These lateral non-pars clefts can rarely be visualized on plain roentgenograms. Plain stress radiographs can demonstrate intervertebral instability at these affected vertebral levels. However, computed tomography (CT) scans give the most accurate picture regarding the extent of such bony defects, as seen in Figures 1-2.

Chronic stress fractures usually present with rounded, sclerotic margins in contrast to acute failures [7]. In order to understand the actual phase of healing and plan the appropriate treatment, magnetic resonance imaging (MRI) is, in fact, a better modality [11]. On the basis of MRI scan [12], spondylolysis has been traditionally graded as: stress reaction (grade 1); incomplete stress fracture (grade 2); acute complete pars fracture (grade 3); and chronic complete pars fracture (grade 4). Similarly, the reparative phases of lytic defects of the pedicle and lamina may also be classified, based on MRI appearance, as: acute healing phase with hypointense marrow on T1 weighted image (WI) and hyperintense signal on T2WI and short T1 inversion recovery (STIR) sequences; or chronic hypertrophic fibrous union with hypointense T1 and T2WI signals, and hypertrophied pedicle morphology. Thus, based on MRI appearance, the lateral nonspondylolytic clefts of the lumbar arch can be classified into four different groups: 1) acute healing pediculolysis; 2) chronic pediculolysis with fibrous nonunion; 3) acute healing laminolysis; and 4) chronic laminolysis with fibrous nonunion. MRI scan can also help in assessing the status of central and foraminal stenosis, and root compression. Scintigraphy is another modality that can indicate the acuteness of the lesion [13]. Active reparative mechanism within the lesions may be demonstrated by enhanced uptake in the pedicle marrow.

### Management

Five major factors that need to be considered in the treatment of these vertebral lesions include: 1) age of the patient; 2) underlying systemic pathology contributing to insufficiency fractures; 3) physical demand (based on the activity level of the patient); 4) chronicity of the lesion (as demonstrated on MRI scan); and 5) degree of intervertebral instability. Acute pediculolysis and laminolysis lesions with hyperintense morphology on MRI are known to respond well to conservative management. The majority of these lesions have been typically described in adolescent (skeletally immature) athletes. A trial of conservative treatment for a period of three to six months, including relative rest and abstinence from sports activities, should definitely be considered in these individuals before any option of surgical intervention is conceived. Some authors also advocate lumbar bracing to enhance healing [13]. Conservative management with orthosis and concomitant anti-osteoporotic medical therapy has also been successfully employed in the management of acute osteoporotic pedicle fractures [10].

However, in these chronic lesions arising out of repetitive stress injuries, one should understand the existence of inherent instability, which has, in fact, led to the development of fibrous, hypertrophic nonunion. These patients, when symptomatic, definitely need to undergo surgical intervention for healing of bony defects [13-14]. The surgical options described in literature for such lesions include direct repair of the lytic lesion [2,15], posterolateral fusion, single- or double-level transforaminal or posterior lumbar interbody fusion (TLIF or PLIF) [10]. In young athletes with chronic pediculolysis without evidence of intervertebral instability, direct repair of the pedicular cleft using compression screws has been described as a treatment option [2,15]. Such motion-preserving surgeries may obviate the need for segmental fusion procedures in patients with high physical activity. However, any evidence of intervertebral instability or significant degenerative spondylosis precludes such a conservative approach. These patients definitely benefit from intervertebral fusion procedures. The surgical management in chronic laminolysis (L5) with contralateral spondylolysis (L5) should be a single-level (L5-S1) transforaminal interbody fusion. However, the levels of vertebra that need to be fused in chronic pediculolysis have remained controversial [5-7,10].

**Figure 3:**
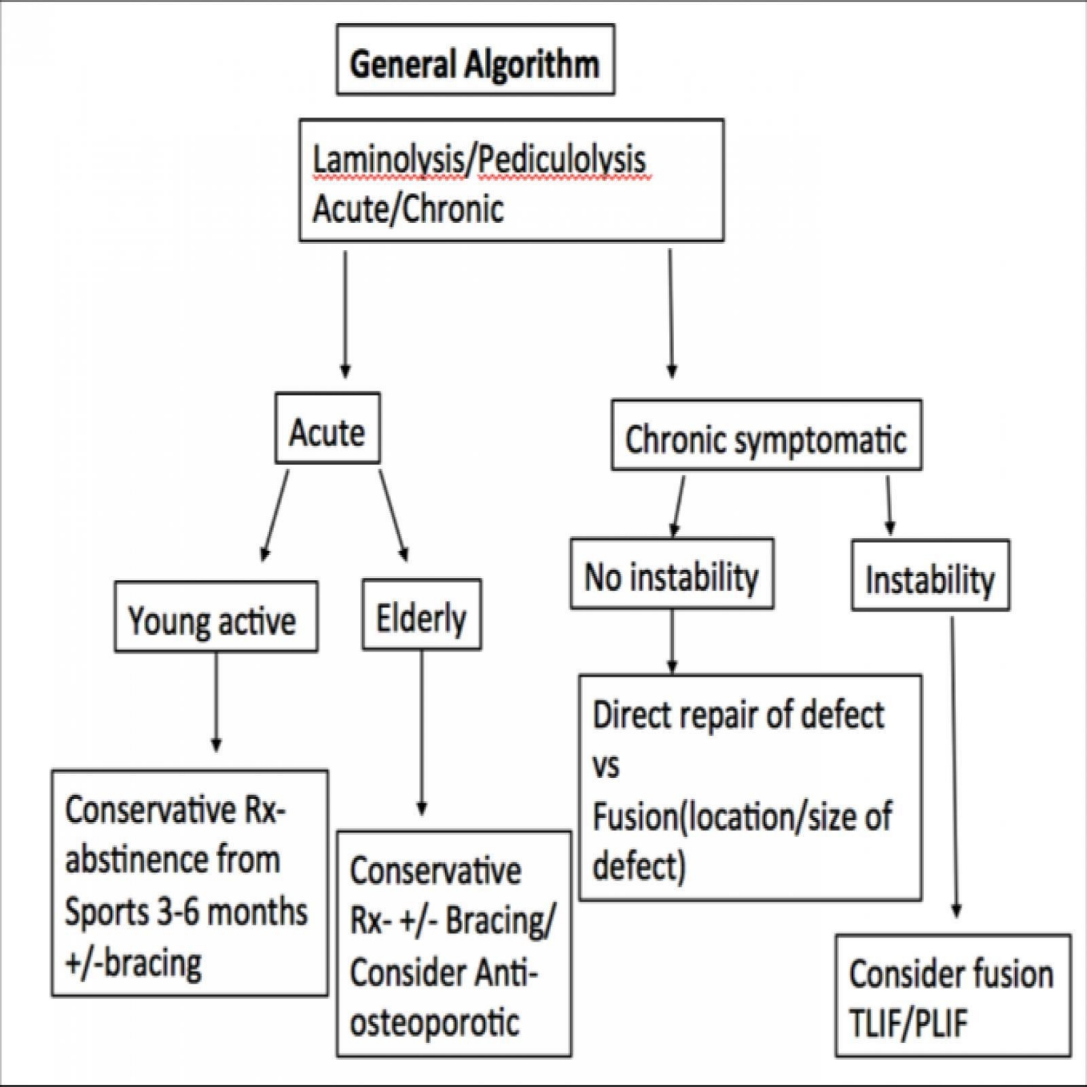
Illustrative algorithm

Most of the literature evidence regarding these nonspondylolytic lesions describes early fractures in adolescent athletes. Since these lesions are extremely uncommon, thorough preoperative evaluation and planning are of utmost relevance to ensure timely diagnosis (when conservative management can be considered) and appropriate treatment. In patients with L5 pediculolysis, who undergo surgical fusion, the need to extend the level of fusion proximally to the L4 pedicle has to be cautiously assessed. Some patients present with a significantly hypertrophied L5 pedicle, where complete decompression of the L5 nerve root may necessitate partial removal of the pedicle itself. As a result, the fixation may need to be extended proximally by one level. We have given a general algorithm below (Figure 3), though the treatment should be based on each patient's specific pathology and clinical findings.

## Conclusions

Good preoperative planning and careful understanding is of utmost importance in the management of these rare lateral nonspondylolytic lesions. In carefully selected patients, both conservative and surgical management can give good outcomes. A vital part of surgical planning includes the identification of ideal candidates for isolated repair of the defect, or single- or double-level fusions. We believe that the guidelines thus purported can help in making the critical distinction between these different patient groups and aid in providing better results in such rare scenarios.
